# Machine Learning-Assisted Square-Spot Laser Surface Reshaping for Sidewall Roughness Control of LDED Ti-6Al-4V Thin-Walled Structures

**DOI:** 10.3390/ma19163406

**Published:** 2026-08-11

**Authors:** Wenjun Yu, Fei Li, Yanze Wang, Pengpeng Xiong, Xiaohu Guan, Feiyue Lyu, Jicheng Chen

**Affiliations:** 1 Chengdu Aircraft Industrial (Group) Co., Ltd., Chengdu 610091, China; 15214725692@163.com (W.Y.); leoscf132@163.com (F.L.); lfy2019ab@163.com (F.L.); 2 College of Materials Science and Technology, Nanjing University of Aeronautics and Astronautics, Nanjing 211106, China; wangyanze512@nuaa.edu.cn (Y.W.); xiongpp76@163.com (P.X.); guanxiaohu@nuaa.edu.cn (X.G.)

**Keywords:** laser directed energy deposition, thin-walled structures, surface quality, square-spot laser reshaping, machine learning

## Abstract

**Highlights:**

- Square-spot laser reshaping reduced sidewall roughness in laser directed energy deposition (LDED) Ti-6Al-4V thin walls.
- An 80-sample process dataset linked laser parameters to sidewall roughness.
- Random forest (RF) outperformed support vector regression (SVR) and eXtreme Gradient Boosting (XGBoost), with R^2^ = 0.940 and root mean square error (RMSE) = 1.760 μm.
- Validation reduced Ra from 28.98 μm to 5.76 μm with an 80.1% roughness reduction.

**Abstract:**

Laser directed energy deposition (LDED) can fabricate Ti-6Al-4V thin-walled structures efficiently, but the deposited sidewalls usually contain adhered particles, layer steps, and waviness that limit surface quality. This study combined square-spot laser surface reshaping with machine learning-assisted parameter design to control sidewall roughness. Sixteen single-factor experiments were first conducted to clarify the effects of laser power, scanning speed, spot overlap ratio, and scan number. An 80-sample dataset was then established to train and compare random forest (RF), support vector regression (SVR), and eXtreme Gradient Boosting (XGBoost) models, and SHapley Additive exPlanations (SHAP) were used to interpret feature contributions. RF showed the best predictive performance, with R^2^ = 0.940 and RMSE = 1.760 μm, and was coupled with Bayesian optimization (BO) for inverse parameter design. For a target arithmetic mean roughness (Ra) of 5 μm, the optimized condition was 500 W, 2.57 mm/s, 48.09% overlap, and five scans. The predicted Ra was 5.02 μm, while the validation experiment yielded 5.76 μm, reducing the initial roughness from 28.98 μm by 80.1%. These results demonstrate that interpretable machine learning can support target-driven square-spot laser reshaping for LDED Ti-6Al-4V thin-walled structures.

## 1. Introduction

Thin-walled metallic components are widely used in aerospace structures such as aero-engine blades, internal flow parts, and integrated channels because they combine low weight, high specific strength, and high material utilization [1,2]. Ti-6Al-4V is one of the most widely used titanium alloys for such structures owing to its high specific strength, corrosion resistance, and elevated-temperature performance [2]. However, the low stiffness of thin walls and the poor machinability of Ti-6Al-4V make conventional subtractive processing difficult and costly [3].

Additive manufacturing provides an effective route for producing complex thin-walled titanium alloy components, and laser directed energy deposition (LDED) is particularly attractive because of its high process flexibility, relatively low material waste, and suitability for near-net-shape fabrication [4,5]. Nevertheless, the thermal boundary condition of a thin wall is highly constrained during layer-by-layer deposition. Heat is mainly dissipated through the previously deposited wall and the substrate, which promotes heat accumulation and makes melt-pool behavior unstable. In addition, the divergence of the gas–powder flow, the instability of laser–powder coupling, and the layer-step effect can easily lead to semi-molten powder adhesion on the sidewall [5,6].

Consequently, as-deposited LDED thin-wall sidewalls usually exhibit adhered particles, irregular protrusions, and step-like waviness, and post-processing is required before they can satisfy precision-assembly or surface-quality requirements. Conventional contact-based finishing is unsuitable for low-stiffness thin walls because mechanical loading can induce deformation and vibration. By contrast, laser surface reshaping is a non-contact method that remelts the near-surface layer and uses surface-tension-driven molten-metal flow to smooth peaks and fill valleys [7]. Recent studies on laser polishing and laser remelting have confirmed that such processes can effectively reduce roughness and improve the surface state of additively manufactured metallic parts, including Ti-6Al-4V and LDED alloys [8,9,10].

Laser surface reshaping is strongly parameter dependent. Previous studies have shown that energy density, laser power, scanning speed, and track overlap all influence the final roughness and remelted surface morphology [11,12]. In particular, the overlap between adjacent scan tracks is critical for controlling boundary grooves and improving overall flatness during multi-track processing [13]. These observations indicate that the final sidewall quality is governed not by a single variable, but by the coupled effects of heat input, interaction time, track coupling, and repeated remelting.

For thin-walled LDED components, two additional constraints are especially important. First, sidewall morphology is multi-scale in nature: adhered particles, local protrusions, and inter-layer waviness coexist on the same surface, so the finishing process must suppress both particle-scale roughness and track-scale undulations. Second, the acceptable processing window is narrow because sidewall smoothing should be achieved without compromising the dimensional integrity of a low-stiffness wall.

The square-spot strategy is therefore not only a geometric choice, but also a process-control choice. Compared with a concentrated Gaussian-like distribution, a flat-top square spot is better suited to multi-track sidewall treatment because it reduces center overheating, improves boundary remelting, and makes the overlap ratio a more effective control variable. Accordingly, roughness reduction depends on coordinated heat input, spreading time, and repeated remelting rather than on any single parameter.

For this reason, trial-and-error optimization alone is inefficient when a target roughness must be achieved. Machine learning has increasingly been used for quality prediction and process design in additive manufacturing because it can capture nonlinear relationships between process variables and output responses [14,15,16,17]. In the present study, random forest (RF), support vector regression (SVR), and eXtreme Gradient Boosting (XGBoost) were used to establish the process-roughness relationship, SHapley Additive exPlanations (SHAP) were used to interpret feature contributions, and Bayesian optimization (BO) was employed for inverse parameter design [18,19,20,21].

Despite the progress of LDED and laser polishing studies, several issues remain unresolved for high-accuracy thin-walled structures. Guidance on fine-scale sidewall formation is still limited, especially for walls processed within narrow thermal windows. In addition, open-loop parameter adjustment is insufficient when geometric instability accumulates during deposition, while conventional trial-and-error optimization is too slow to identify both a feasible processing window and a target-oriented optimum.

Accordingly, this study focused on sidewall roughness control in LDED Ti-6Al-4V thin-walled structures by combining square-spot laser surface reshaping experiments with machine learning-assisted optimization. The novelty of the work is that square flat-top sidewall reshaping, interpretable roughness prediction, and Bayesian inverse design are integrated into one validated workflow for target roughness control. Different from conventional empirical laser-polishing parameter screening, the proposed route links single-factor physical trends, SHAP-based model interpretation, BO-based parameter recommendation, and experimental validation for thin-walled LDED sidewalls.

## 2. Materials and Methods

### 2.1. Materials and Powder Pretreatment

Ti-6Al-4V (TC4) titanium alloy plates were used as the substrates for laser directed energy deposition (LDED). The substrate size was 150 mm × 150 mm × 10 mm. The nominal chemical composition of the substrate was 6.04 wt.% Al, 3.71 wt.% V, ≤0.15 wt.% Fe, ≤0.10 wt.% C, ≤0.05 wt.% N, ≤0.20 wt.% O, and Ti balance. Gas-atomized Ti-6Al-4V powder with a particle-size range of 15–53 μm was used as the feedstock. Before deposition, the powder was dried in a vacuum oven at 102 °C for 8 h to remove adsorbed moisture. After furnace cooling to room temperature, the powder was used within 24 h to reduce secondary moisture absorption.

The powder pretreatment was included because Ti-6Al-4V powder is chemically active and can absorb moisture during storage. Adsorbed moisture may reduce powder flowability, increase the risk of powder-feeding instability, and promote pore or oxide formation during laser processing. The drying and short storage interval were therefore used to keep the feedstock condition consistent before deposition.

Before LDED, the substrate surface was mechanically ground to remove oxide scale and surface contamination. The surface was then wiped with non-woven cloth to remove metallic debris and residual powder, followed by repeated cleaning with anhydrous ethanol. Deposition was carried out within 24 h after cleaning and drying.

### 2.2. LDED Fabrication of Thin-Walled Structures

**Figure 1:**
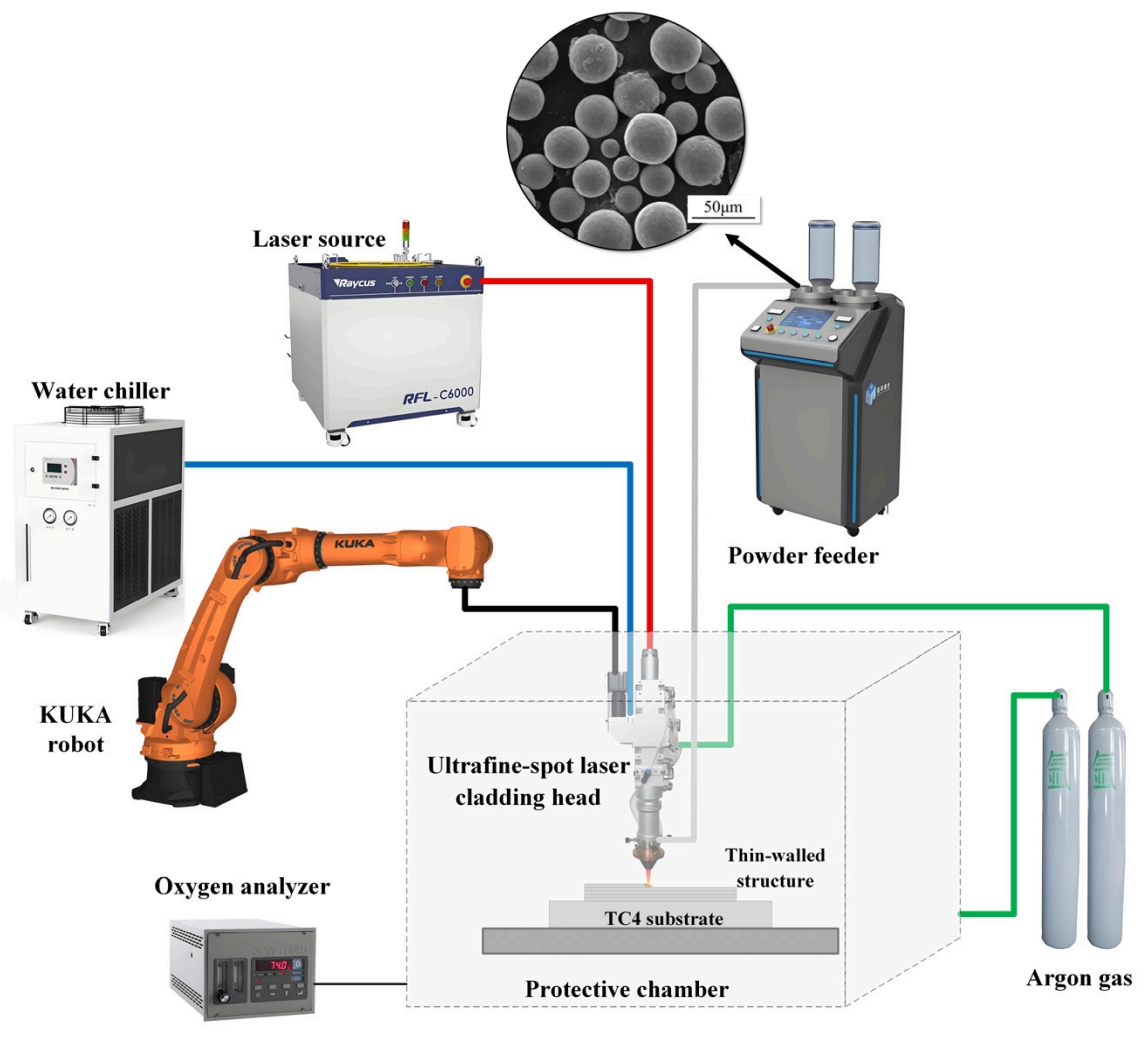
LDED fabrication platform.

The custom-integrated LDED system consisted of a Raycus RFL-C6000 fiber laser (Wuhan Raycus Fiber Laser Technologies Co., Ltd., Wuhan, China), an industrial water chiller, a KUKA KR 70 R2100 six-axis robot (KUKA Deutschland GmbH, Augsburg, Germany), an ultrafine-spot laser cladding head, a turntable powder feeder, an oxygen analyzer, and a sealed processing chamber (Figure 1). The laser had a maximum power of 6000 W, a central wavelength of 1080 ± 5 nm, a fiber core diameter of 25 μm, and a power-adjustment range of 10–100%. The ultrafine LDED laser spot diameter was 0.8 mm. During deposition, high-purity argon (≥99.99%) was continuously introduced into the sealed chamber, and the oxygen concentration was kept below 200 ppm.

The sealed chamber and oxygen monitoring were particularly important for titanium alloy processing because Ti-6Al-4V is sensitive to oxidation at elevated temperature. Maintaining a low-oxygen argon atmosphere helped reduce oxidation during both deposition and subsequent surface treatment, improving the repeatability of the morphology and roughness measurements.

Thin-walled Ti-6Al-4V samples for the subsequent sidewall surface-reshaping experiments were fabricated using the optimized LDED parameters obtained in the preceding process study: a laser power of 800 W, a scanning speed of 30 mm/s, and a powder feeding rate of 4.14 g/min. The adaptive defocus correction was performed every 10 deposited layers.

### 2.3. Square-Spot Laser Surface Reshaping

**Figure 2:**
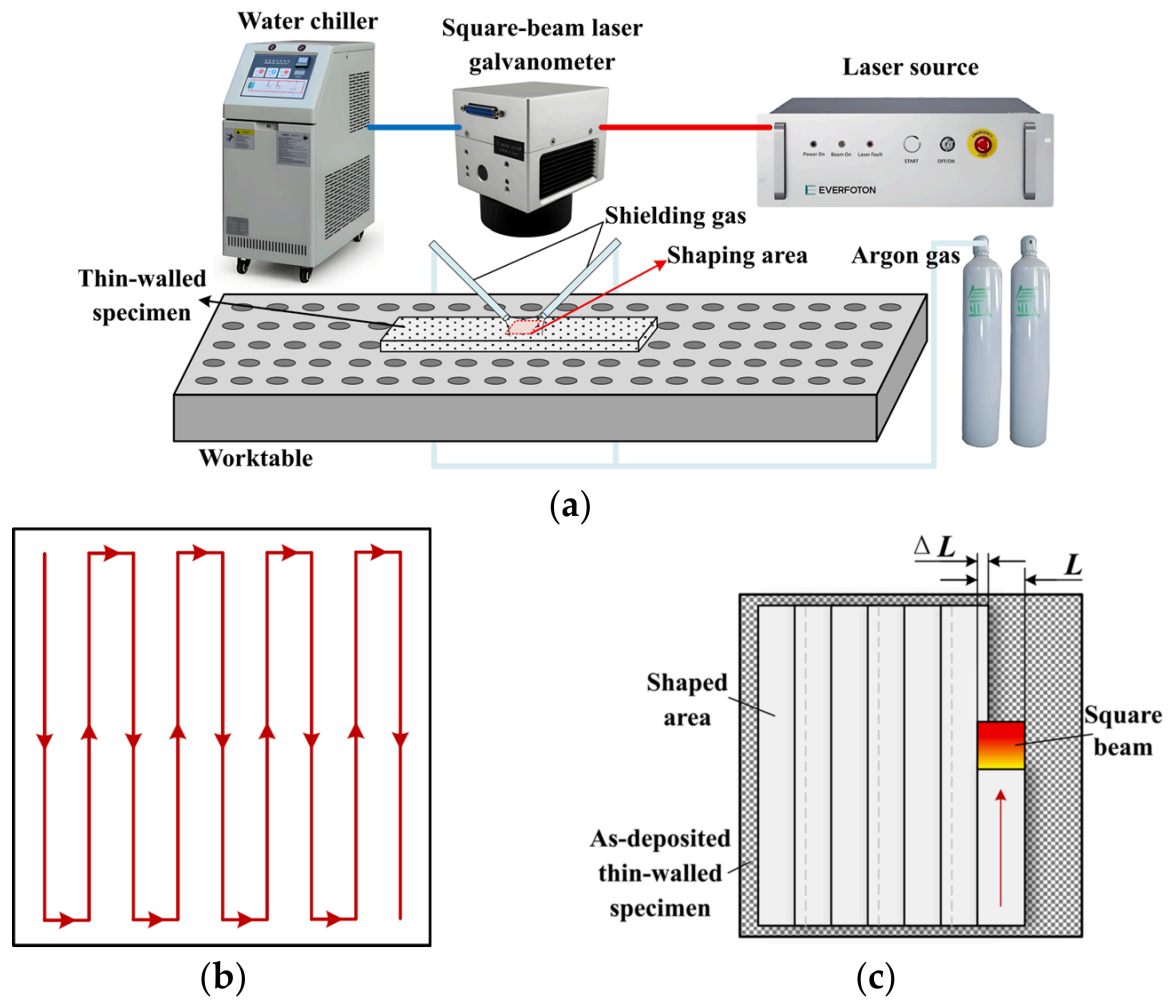
Square-spot laser reshaping system and scanning principle: (**a**) schematic of the square-spot laser reshaping system; (**b**) zigzag scanning path; (**c**) overlap-ratio definition and reshaped area.

**Figure 3:**
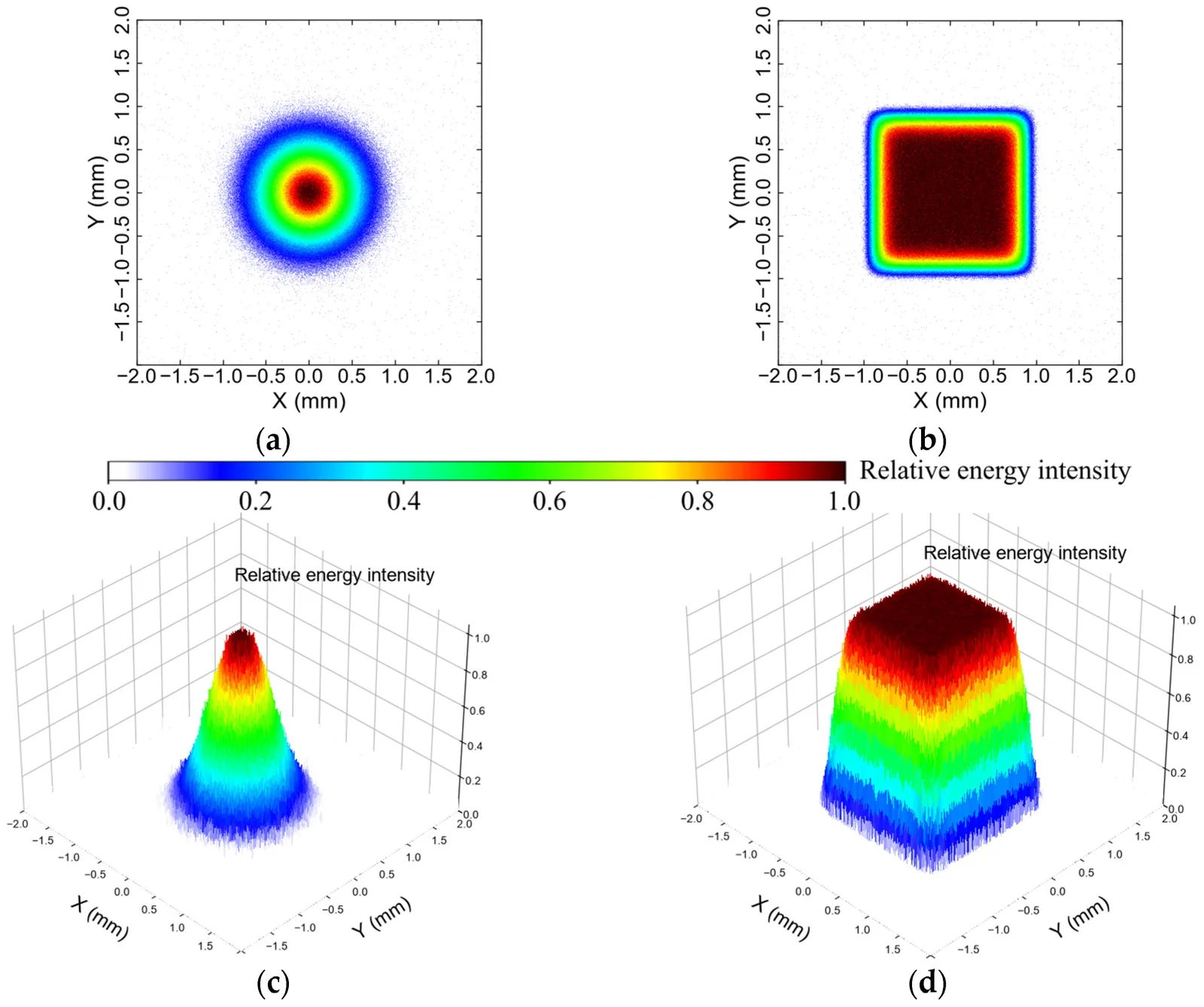
Energy-distribution comparison between Gaussian and square flat-top laser spots: (**a**) two-dimensional Gaussian energy distribution; (**b**) two-dimensional square flat-top energy distribution; (**c**) three-dimensional Gaussian energy distribution; (**d**) three-dimensional square flat-top energy distribution.

The sidewall surface reshaping was performed using a square-spot laser galvanometer system (Figure 2). The system included a laser source, water chiller, square-spot galvanometer, argon shielding gas path, and worktable. The galvanometer modulated the fiber laser beam into a square flat-top spot with a side length of 0.1 mm. The available laser-power range was 0–500 W. A zigzag scanning path was used to improve the uniformity of surface heat input. The spot overlap ratio was defined as the ratio between the overlap width of two adjacent scanning tracks and the side length of the square laser spot: *O* = (*w_o_*/*s*) × 100% = [(*s* − *h*)/*s*] × 100%, where O is the overlap ratio, wo is the overlap width, s is the square-spot side length, and h is the center-to-center spacing between adjacent scanning tracks. The energy-distribution contrast between the Gaussian and square flat-top spots is shown in Figure 3.

The square-spot energy distribution was compared with a Gaussian distribution before the reshaping experiments. In a Gaussian beam, the central energy density is much higher than the edge energy density, so the processed surface may simultaneously experience center overmelting and edge underheating. In a square flat-top beam, the energy distribution inside the spot is flatter, and the boundary of the effective processing area is more regular. This makes the overlap ratio a physically meaningful control variable for maintaining uniform remelting between adjacent scan tracks.

Four reshaping variables were investigated: laser power, scanning speed, spot overlap ratio, and scan number. The single-factor experimental matrix contained 16 groups, as listed in Table 1. These experiments were used to identify the effects of each parameter on sidewall morphology and Ra. The repeated condition of 500 W, 2 mm/s, 0% overlap, and one scan served as the common baseline for different single-factor series; these repeated entries indicate the baseline setting used when another parameter was varied, rather than independent replicate experiments.

### 2.4. Surface Characterization

After LDED and surface reshaping, the samples were cleaned with ethanol to remove surface oil and dust. The sidewall morphology and roughness were measured using a VK-X1000 confocal laser scanning microscope (CLSM; KEYENCE Corporation, Osaka, Japan). The measured response was the arithmetic mean roughness Ra. For the single-factor tests, a 3 mm × 3 mm measurement region was selected across the reshaped/unreshaped boundary, with the two regions occupying approximately half of the field.

The confocal microscope was used to acquire both optical surface images and three-dimensional height maps. Thus, the analysis combined quantitative Ra values with morphology evolution, allowing the roughness trend to be interpreted together with particle remelting, molten-layer spreading, track-boundary formation, and repeated-scan leveling.

### 2.5. Dataset Construction and Machine-Learning Models

The process-roughness dataset contained 80 samples, as listed in Table 2. The input variables were laser power, scanning speed, spot overlap ratio, and scan number, and the output variable was Ra. The dataset combined 16 orthogonal-design baseline samples with 64 additional parameter combinations selected using Latin hypercube sampling (LHS), which improved coverage of the multidimensional parameter space before roughness measurement. The Ra values in the dataset ranged from 1.82 μm to 27.20 μm.

The mixed sampling strategy was selected to balance statistical reliability and spatial coverage. The 16 orthogonal-design samples provided balanced factor-level information for estimating main effects and key interactions, whereas the LHS samples filled the parameter space between the discrete experimental levels. This design was intended to improve the model sensitivity to nonlinear coupling among heat input, interaction time, track overlap, and repeated remelting.

Three regression models were compared: random forest (RF), support vector regression (SVR), and eXtreme Gradient Boosting (XGBoost). RF was based on bootstrap aggregation and classification and regression tree (CART) regressors. SVR used a Gaussian kernel function. XGBoost was based on gradient-boosted decision trees with regularized objective optimization. Model performance was evaluated using the root mean square error (RMSE), coefficient of determination (R^2^), and mean absolute error (MAE). SHapley Additive exPlanations (SHAP) were then used to interpret the contribution of each process parameter to the predicted Ra.

RMSE was used because large prediction deviations should be strongly penalized in precision surface control. R2 quantified the proportion of roughness variance explained by each model, while MAE provided a direct average error in micrometers. To reduce the risk of overfitting, model performance was evaluated using held-out independent test samples that were not used for model fitting. The reported test-set accuracy therefore reflects independent predictive performance rather than training-set fitting accuracy. In addition, the 80-sample dataset was interpreted as sufficient for local process-window optimization within the tested parameter space, while broader generalization still requires additional data from other systems, wall geometries, and material batches.

### 2.6. Bayesian Optimization and Validation

The best-performing RF regression model was used as the forward roughness predictor and coupled with Bayesian optimization (BO) to inversely determine the process parameters for a target roughness. The BO procedure used a Gaussian-kernel probabilistic surrogate model and an expected-improvement acquisition function to guide sampling. The target Ra was set to 5 μm. The search bounds were 200–500 W for laser power, 1–5 mm/s for scanning speed, 0–50% for spot overlap ratio, and 1–5 for scan number, as listed in Table 3. The optimization was run for a maximum of 50 iterations. The final optimized parameter combination was 500 W, 2.57 mm/s, 48.09% overlap, and five scans, with a predicted Ra of 5.02 μm.

The inverse-design problem was formulated as minimizing the difference between the model-predicted roughness and the target roughness within the experimentally feasible parameter domain. In this framework, BO treats the RF predictor as a black-box objective function and uses the acquisition function to balance exploitation of promising low-error regions with exploration of uncertain regions in the parameter space.

To validate the inverse-design result, a surface-reshaping experiment was conducted using the optimized parameter combination. The as-deposited sidewall Ra was 28.98 μm. After reshaping, the measured Ra decreased to 5.76 μm, corresponding to an 80.1% reduction. The absolute deviation between the predicted and experimental roughness values was 0.76 μm, and the relative error was 14.7%.

## 3. Results

### 3.1. As-Deposited Sidewall Morphology

**Figure 4:**
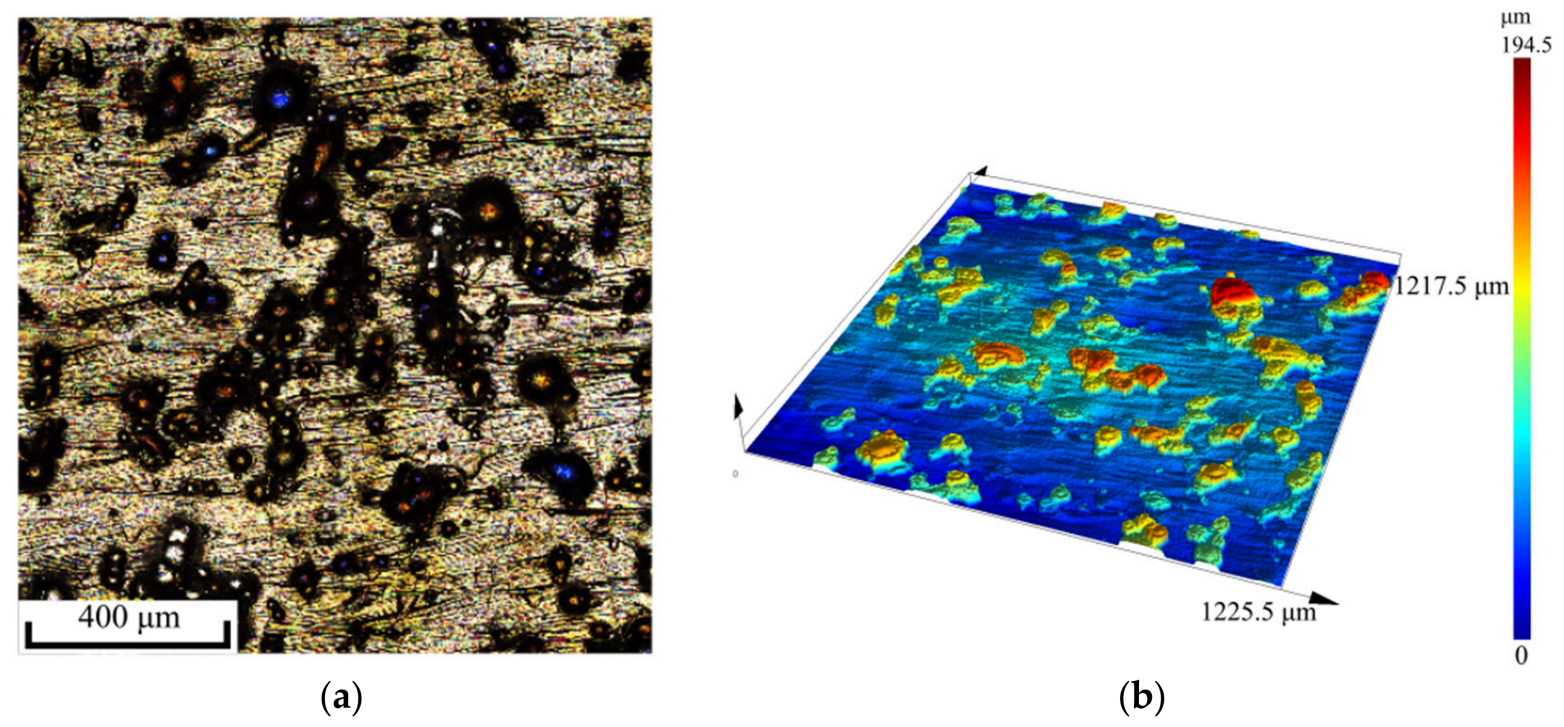
As-deposited sidewall morphology before surface reshaping: (**a**) optical morphology of the as-deposited sidewall; (**b**) three-dimensional height map of the as-deposited sidewall.

Figure 4 shows the typical sidewall morphology of the as-deposited LDED Ti-6Al-4V thin-walled structure before square-spot laser reshaping. The sidewall contained adhered semi-molten powder particles, layer-step features, and irregular height fluctuations. These defects are consistent with the nonequilibrium melting and solidification behavior of the LDED process. They also explain why the as-deposited surface cannot directly meet the requirement for a low-roughness thin-wall sidewall. In the validation sample, the initial Ra reached 28.98 μm, so the as-deposited morphology provided a clear baseline for evaluating the reshaping effect.

This initial morphology can be attributed to the combined effects of powder adhesion, rapid solidification, and layer-by-layer height fluctuation. Semi-molten particles remain attached when the local heat input is insufficient for complete melting, whereas repeated deposition leaves step-like features at the sidewall. These two defect types provide different length-scale roughness components, which must both be reduced during surface reshaping.

### 3.2. Effects of Square-Spot Laser Reshaping Parameters

**Figure 5:**
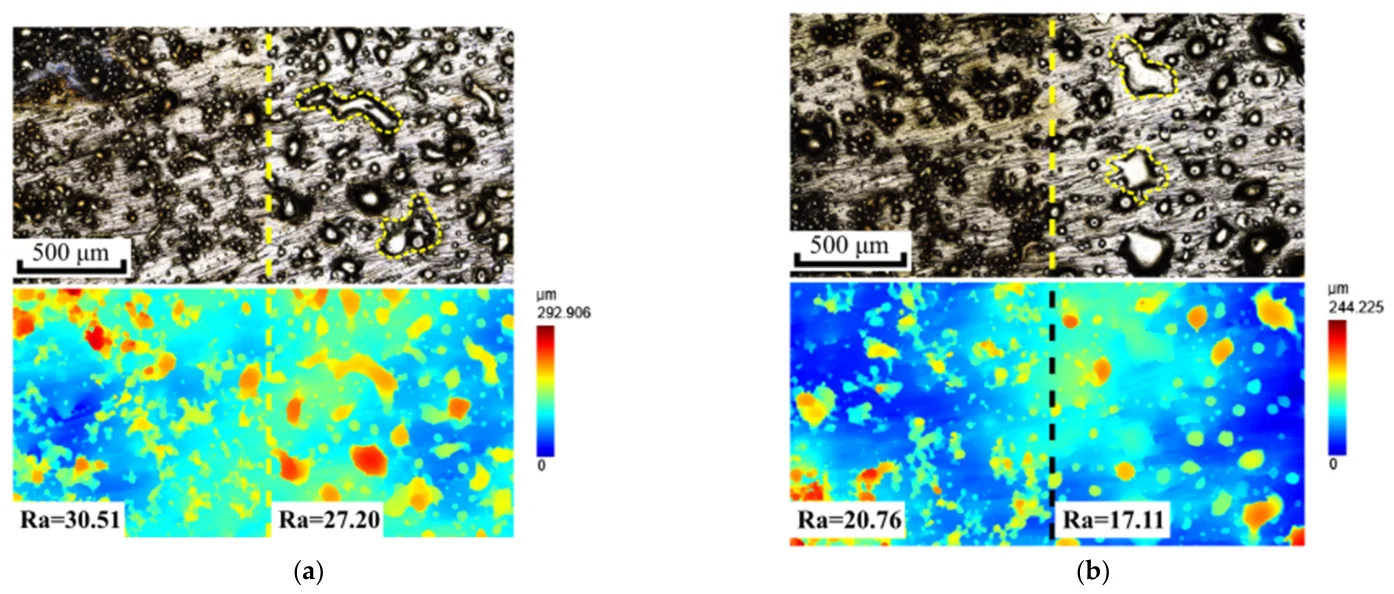

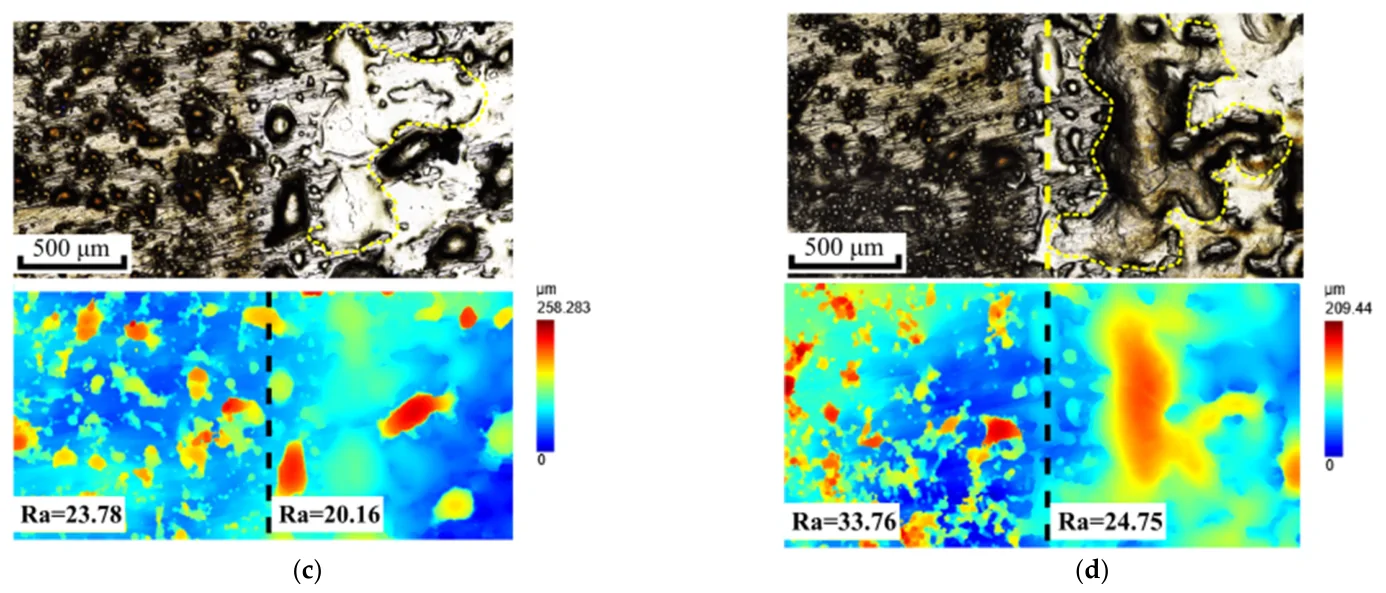
Effect of laser power on sidewall morphology and roughness: (**a**) 200 W; (**b**) 300 W; (**c**) 400 W; (**d**) 500 W.

Figure 5 compares the surface morphology and roughness obtained under different laser powers. At 200 W, the heat input was insufficient to fully remelt the adhered particles, and the surface still contained pronounced peaks and valleys. Increasing the power improved remelting and material spreading. However, excessive local heat input could also induce coarse remelted undulations or local surface damage if it was not balanced by scanning speed, overlap ratio, and scan number. Therefore, laser power supplied the basic energy required for defect removal, but it did not determine the final surface quality by itself.

The power series shows the existence of a processing window rather than a simple monotonic trend. At 200 W, many isolated particles and irregular molten regions remained because the surface layer could not be fully remelted. At 300 W, the number of unmelted particles decreased and the surface became more continuous. At 400 W, most particles were remelted, although local droplets could still form when molten metal did not spread sufficiently before solidification. At 500 W under isolated power variation, excessive energy input produced coarse remelted undulations and local discoloration. Thus, high power is beneficial only when coordinated with scanning speed, overlap ratio, and scan number.

**Figure 6:**
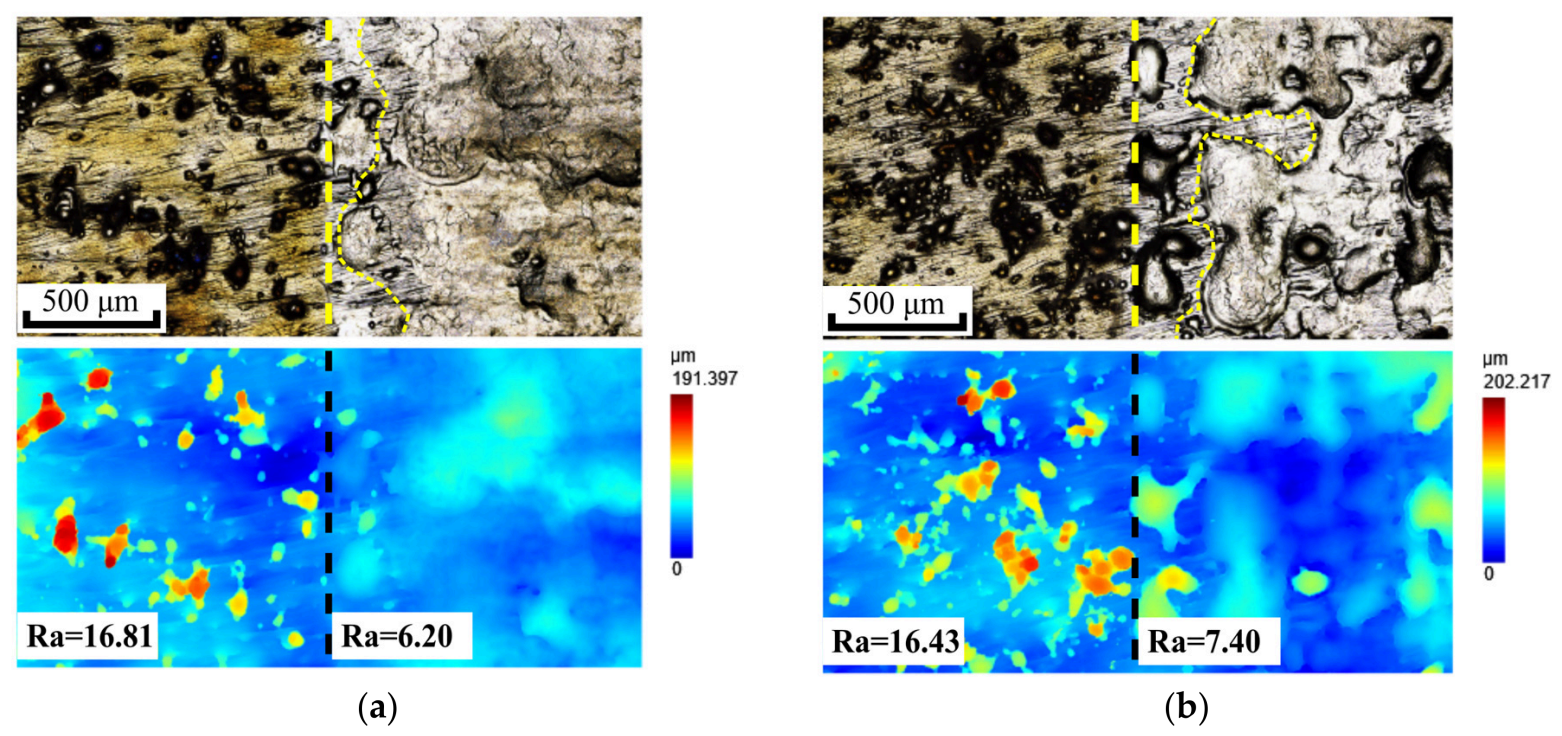

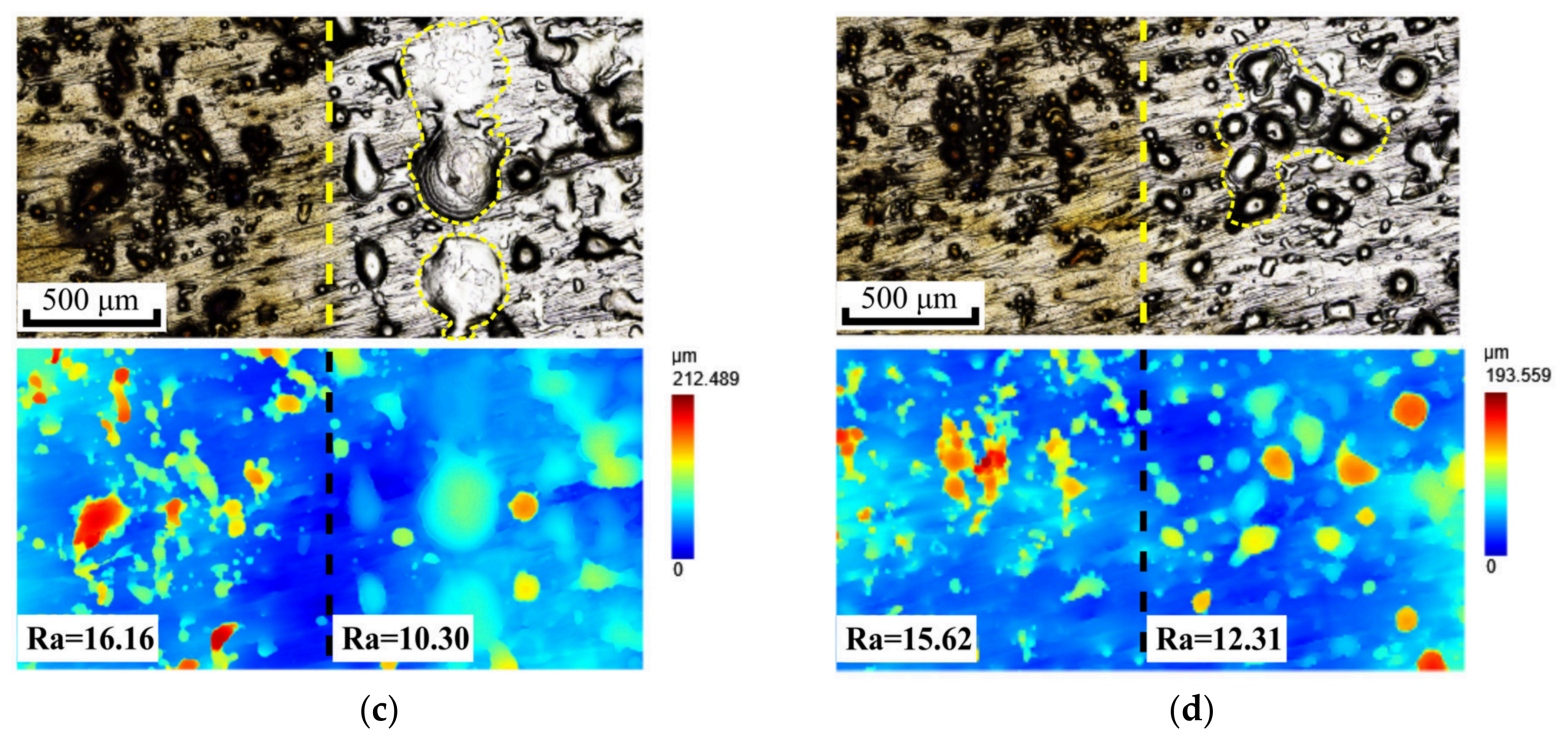
Effect of scanning speed on sidewall morphology and roughness: (**a**) 1 mm/s; (**b**) 2 mm/s; (**c**) 3 mm/s; (**d**) 4 mm/s.

Figure 6 shows the effect of scanning speed. When the scanning speed was 1 mm/s, the longer laser residence time promoted surface melting and viscous flow, reducing Ra to 6.20 μm. As the scanning speed increased to 2 mm/s, Ra increased to 7.40 μm. At 3 mm/s, the lower line energy shortened the flow time of the molten surface layer, and Ra increased to 10.30 μm. At higher speed, the heat input was insufficient for stable remelting, so the surface became rougher. These results show that scanning speed mainly controls the available interaction time and the solidification rate of the reshaped surface layer.

The morphology evolution confirms this interpretation. At low speed, the longer residence time allows a shallow molten pool to form and gives the liquid metal enough time to redistribute. As speed increases, the available line energy decreases, cooling becomes faster, and the molten layer has less time to fill valleys. At 3 mm/s, balling-like protrusions were observed, indicating that the molten metal tended to contract to reduce surface energy when spreading was insufficient.

**Figure 7:**
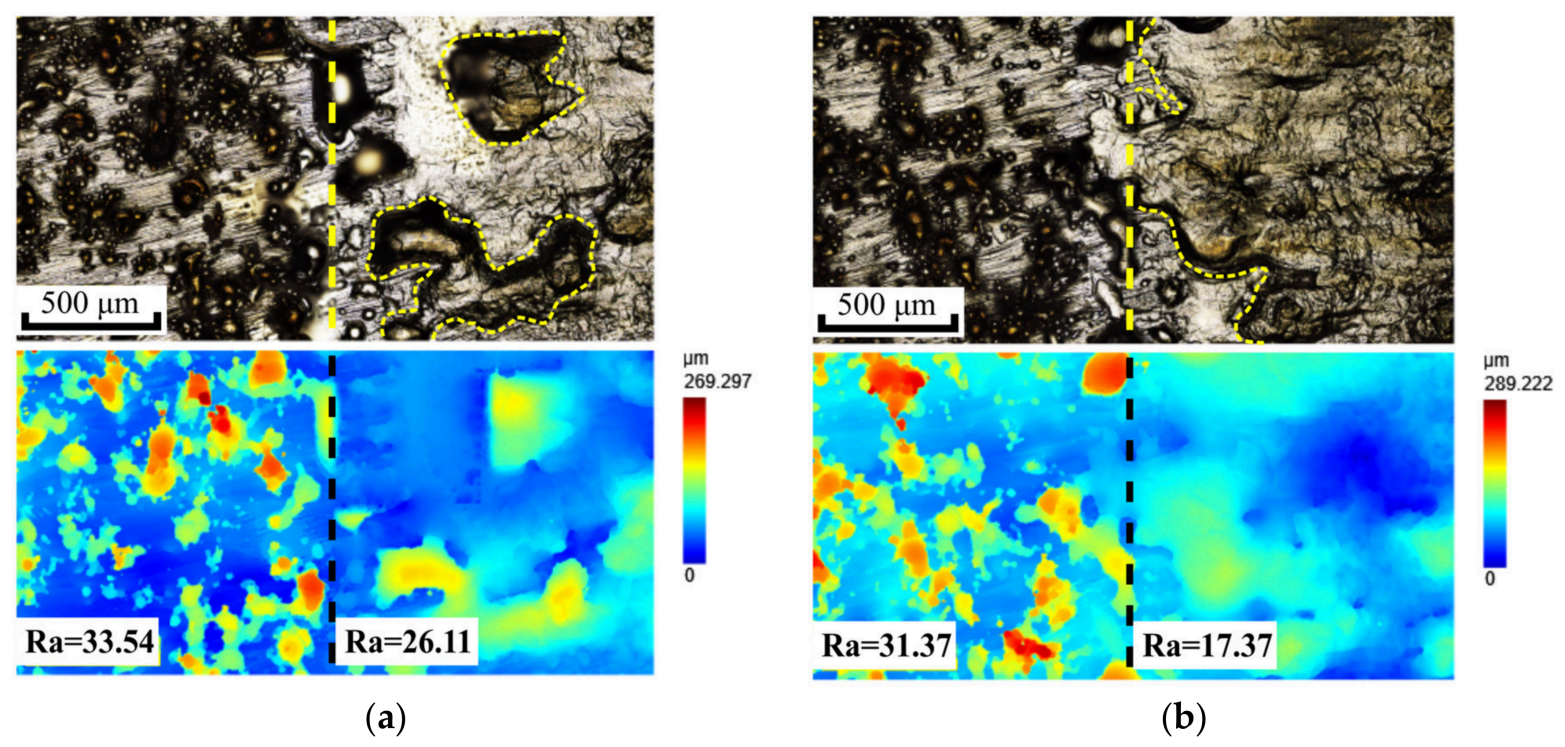

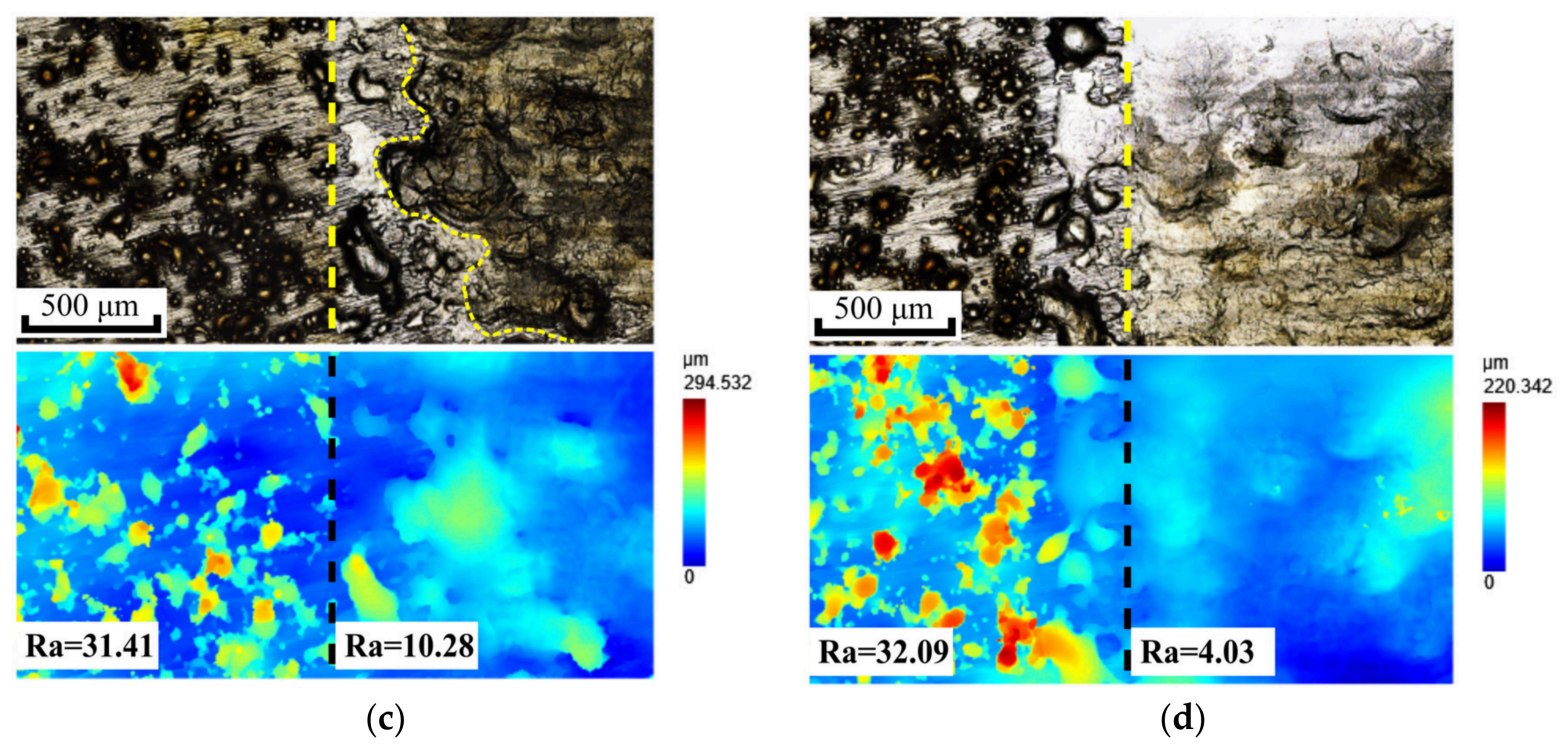
Effect of spot overlap ratio on sidewall morphology and roughness: (**a**) 0%; (**b**) 10%; (**c**) 20%; (**d**) 30%.

Figure 7 presents the effect of spot overlap ratio. At low overlap ratios, the adjacent tracks were weakly coupled, and grooves or incomplete remelting remained between tracks. Increasing the overlap ratio enhanced heat accumulation and track-to-track leveling. When the overlap ratio reached 20%, Ra decreased to 10.28 μm. At 30% overlap, the remelted tracks connected more continuously, and Ra decreased to 4.03 μm, corresponding to an 87.4% reduction relative to the initial surface in that test. This confirms that overlap ratio is essential for suppressing inter-track height differences during square-spot surface reshaping.

At 0% and 10% overlap, adjacent tracks were weakly connected, so grooves and incomplete remelting remained near the track boundaries. When the overlap increased to 20%, the accumulated heat between tracks improved molten-layer continuity. At 30% overlap, the edge of the previous track was sufficiently remelted by the next track, producing a more continuous transition and a much lower Ra. This result supports the use of overlap ratio as a key parameter for square-spot multi-track reshaping.

**Figure 8:**
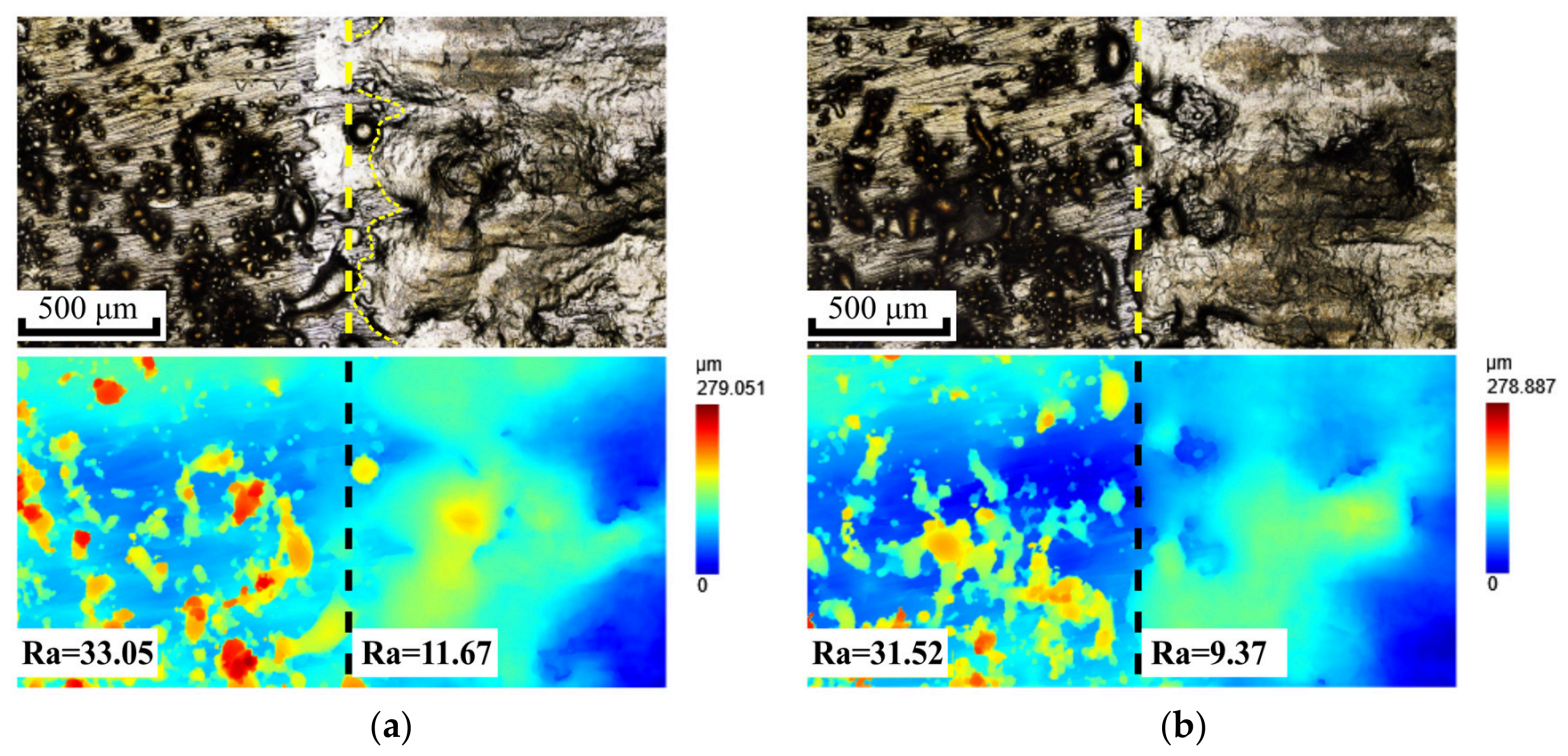

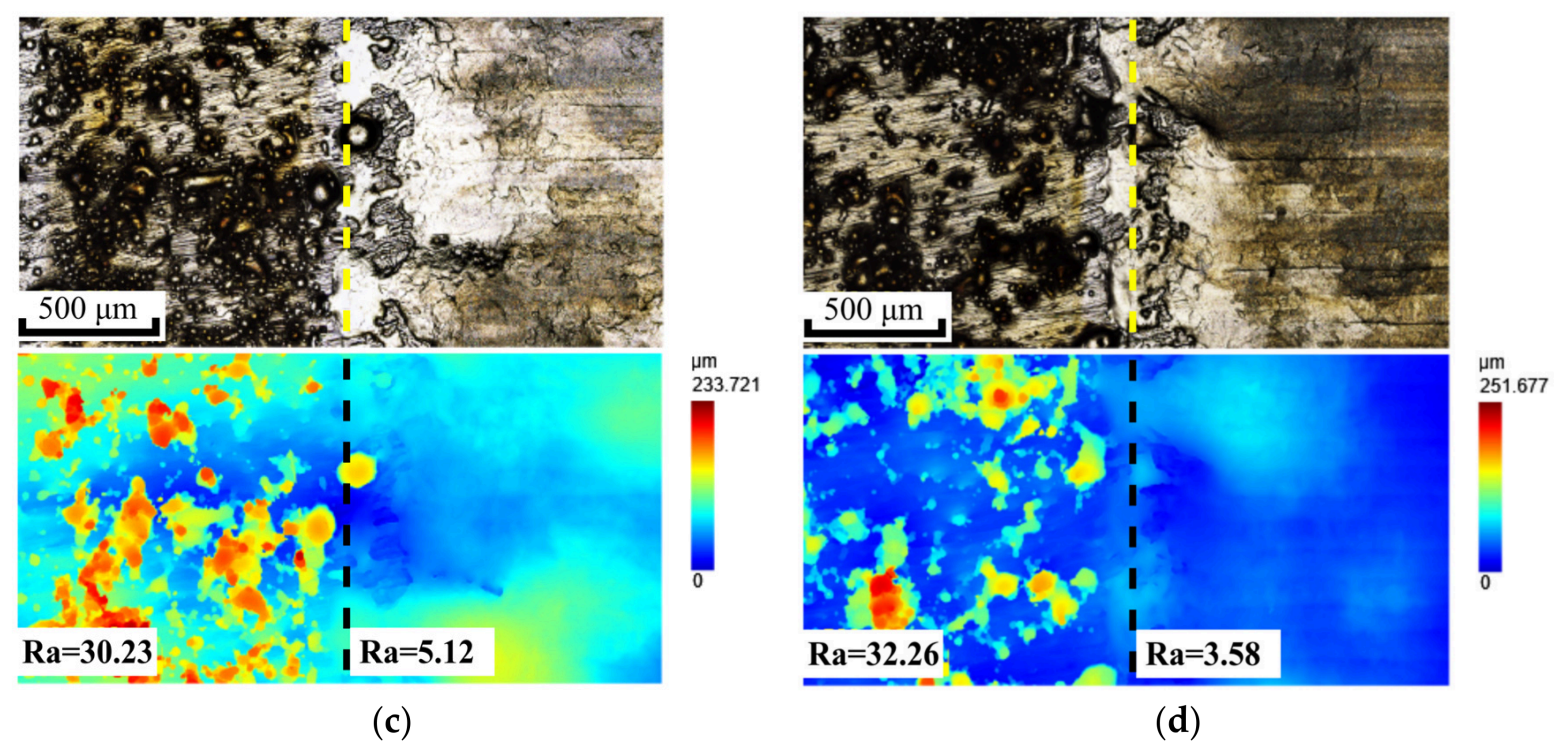
Effect of scan number on sidewall morphology and roughness: (**a**) one scan; (**b**) two scans; (**c**) three scans; (**d**) four scans.

Figure 8 shows the influence of scan number. A single scan reduced Ra to 11.67 μm by removing the highest surface asperities, but some remelted particles and local boundary features remained. With two scans, Ra further decreased to 9.37 μm. Additional scans promoted repeated remelting and leveling of residual surface waves. After four scans, Ra reached 3.58 μm, nearly 90% lower than the initial surface roughness in that group. These results show that repeated scanning can progressively smooth high-frequency roughness through cyclic remelting, although excessive thermal accumulation should still be controlled.

The repeated-scan effect is consistent with a progressive leveling mechanism. The first scan mainly removes the highest asperities, the second scan remelts residual boundary features, and subsequent scans continue to soften and redistribute the remaining surface waves. This explains why four scans produced the lowest Ra in the single-factor group, while also indicating that further increases should be assessed carefully because repeated thermal cycles may increase oxidation or geometric distortion.

### 3.3. Machine-Learning Prediction of Surface Roughness

**Figure 9:**
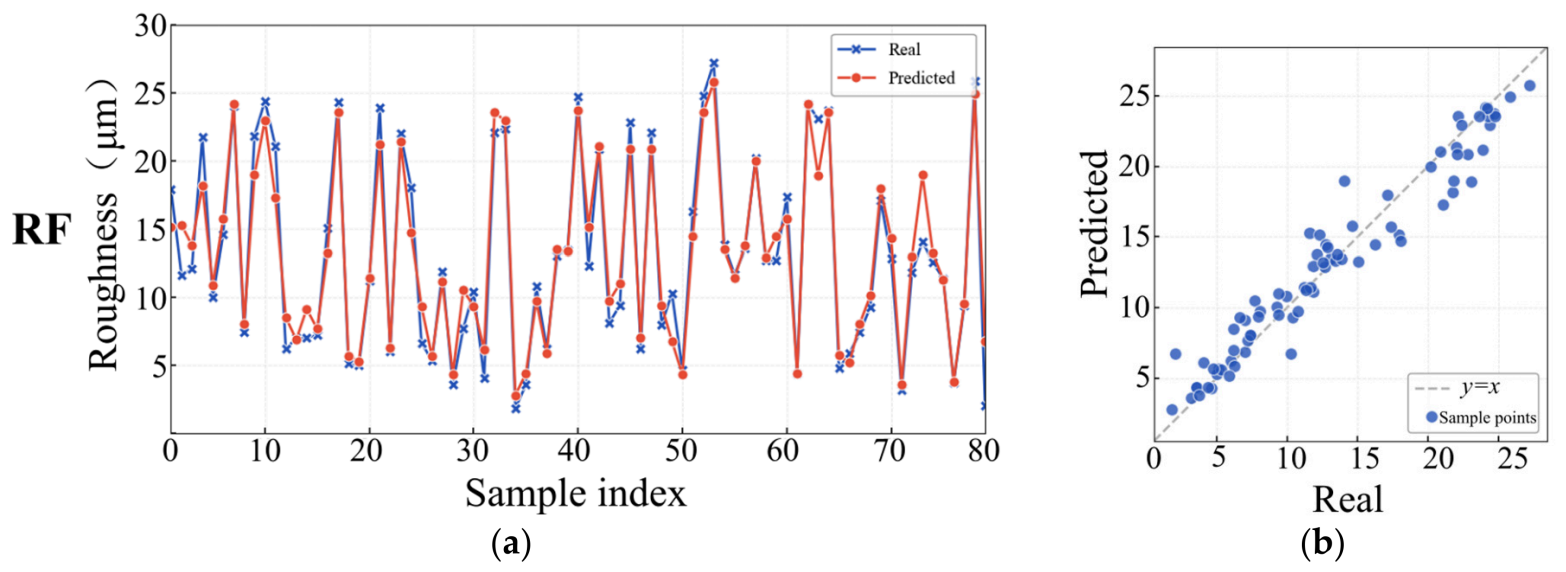

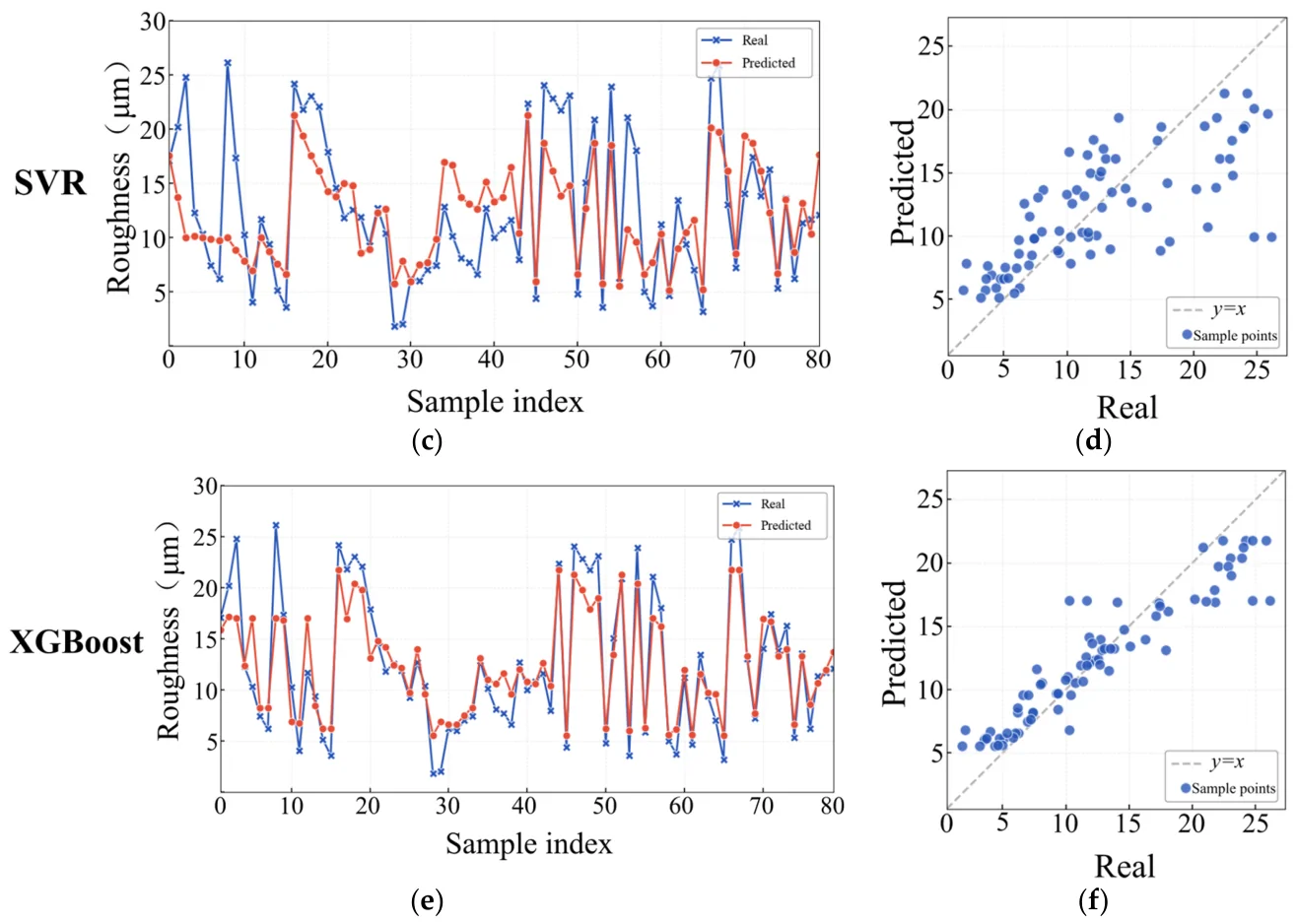
Prediction performance comparison among RF, SVR, and XGBoost: (**a**) RF real and predicted values versus sample index; (**b**) RF predicted-versus-real plot; (**c**) SVR real and predicted values versus sample index; (**d**) SVR predicted-versus-real plot; (**e**) XGBoost real and predicted values versus sample index; (**f**) XGBoost predicted-versus-real plot.

The 80-sample dataset was used to establish the relationship between four reshaping parameters and Ra. The dataset covered Ra values from 1.82 μm to 27.20 μm, indicating a wide response range for model training and testing. Figure 9 compares the prediction performance of RF, SVR, and XGBoost. Among the three models, RF achieved the best overall accuracy, with R^2^ = 0.940 and RMSE = 1.760 μm. XGBoost also captured the nonlinear trend, but its R^2^ was lower at 0.839. SVR showed the weakest predictive capability, with R^2^ = 0.525, indicating that it did not sufficiently capture the local nonlinear changes in the small process dataset.

The difference among the three models reflects their different treatment of local nonlinear behavior. SVR followed the overall trend but produced a smoother response and missed sharp roughness changes at local extrema. XGBoost tracked many local variations, but its residual-fitting strategy may be more sensitive to individual edge samples in a small dataset. RF provided a more stable compromise by averaging multiple decision trees trained on bootstrap samples, which reduced variance while preserving local partitioning of the parameter space.

The superior RF performance can be attributed to the ensemble structure of multiple decision trees. The model can divide the parameter space into local regions and reduce variance through bagging. This behavior is suitable for the present dataset, where roughness changes are not governed by a single monotonic relationship but by coupled effects among heat input, overlap, and repeated remelting. Because the final BO validation experiment was performed at an optimized parameter combination that was not used as the direct training target, it also provided an additional process-level check on the RF-BO workflow.

### 3.4. SHAP Interpretation

**Figure 10:**
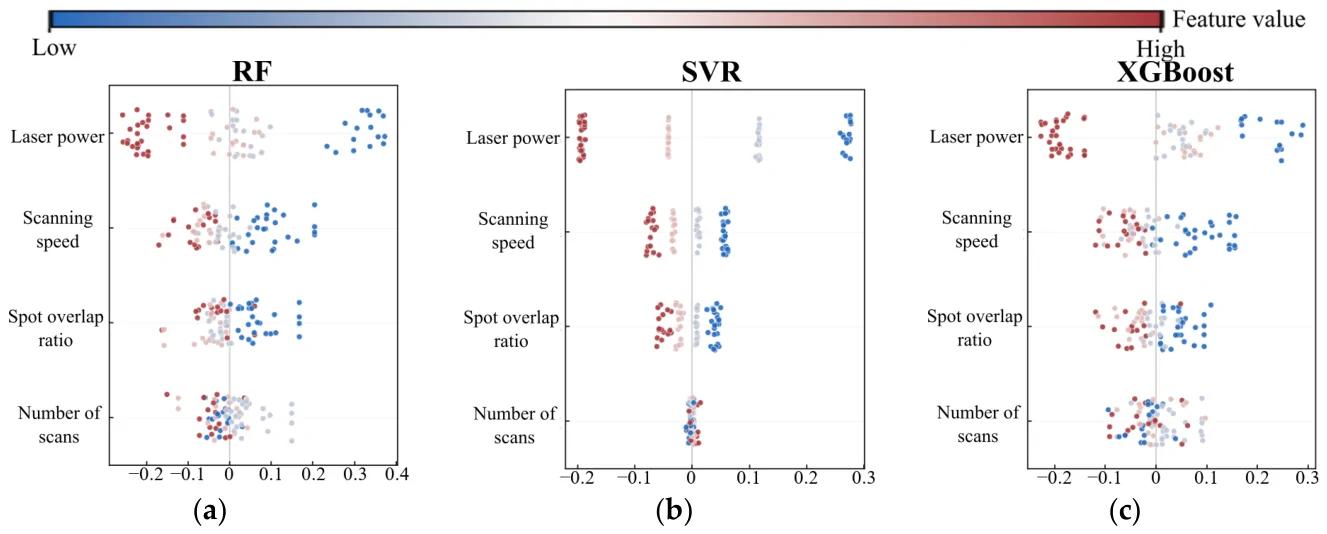
SHAP interpretation of process-parameter contributions: (**a**) RF model; (**b**) SVR model; (**c**) XGBoost model.

Figure 10 gives the SHAP summaries for the three models. Although the models used different regression strategies, the feature-importance ranking was generally consistent. Laser power showed the largest mean absolute SHAP contribution to the predicted roughness. Scanning speed and spot overlap ratio followed, while scan number had a smaller overall contribution. This ranking agrees with the single-factor experiments, where laser power determined whether sufficient remelting could occur, scanning speed controlled residence time, and overlap ratio affected track-to-track leveling.

The SHAP value distribution also provides physical information beyond the global ranking. High laser power generally shifted the model output toward lower predicted roughness, indicating that sufficient energy input promoted remelting and surface leveling. Higher overlap ratios also contributed to roughness reduction because adjacent tracks could remelt and smooth the boundary region more effectively. For RF and XGBoost, higher scan numbers tended to reduce roughness, reflecting the repeated-remelting mechanism observed experimentally. In contrast, the SVR SHAP results were more clustered, suggesting that SVR was less sensitive to local interactions in the discrete process space.

The SHAP scatter patterns also reveal model behavior. For all three models, high-power samples were mainly associated with negative SHAP values, indicating a contribution toward lower predicted roughness, whereas low-power samples tended to increase predicted roughness. Higher overlap ratios produced a similar negative contribution because track coupling improved. In RF and XGBoost, scan number also showed a roughness-reducing contribution at higher values, while in SVR its SHAP values clustered near zero. This suggests that SVR treated scan number as a weak feature and did not fully capture the repeated-remelting effect.

### 3.5. Bayesian Optimization for Target Roughness

**Figure 11:**
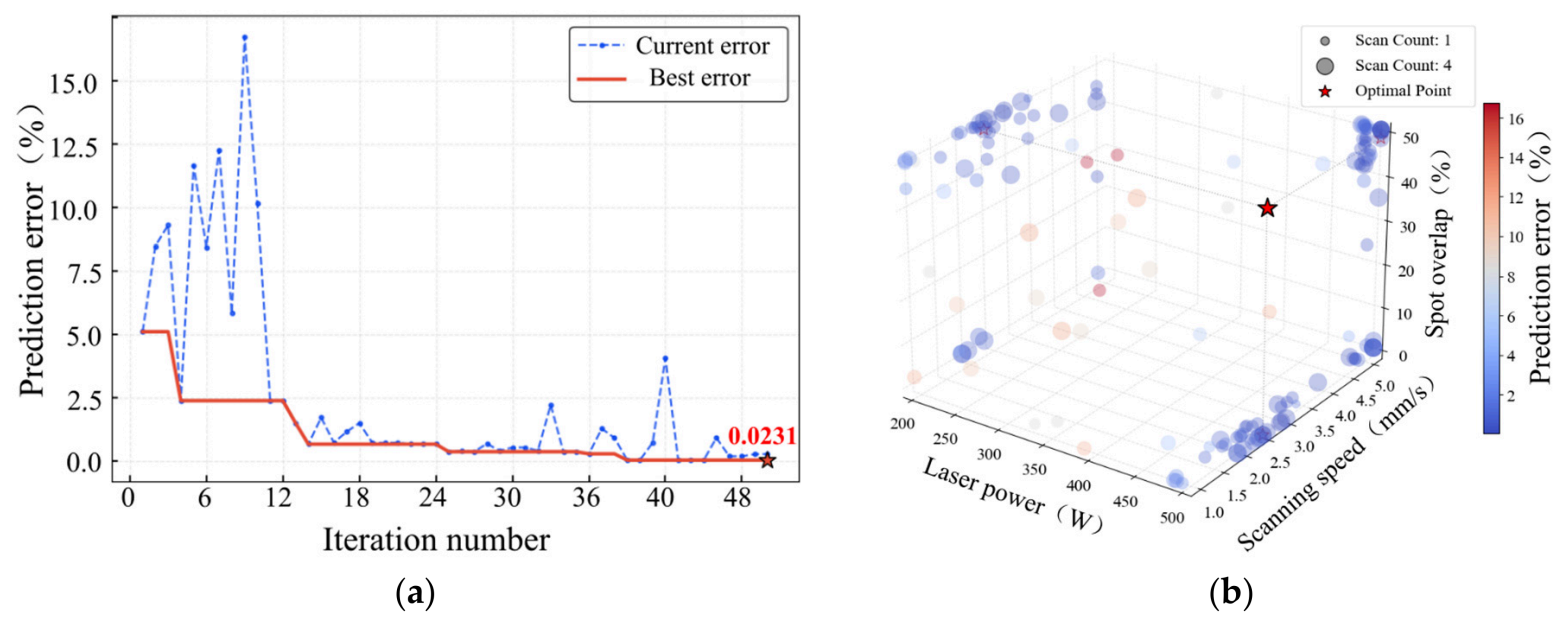
Bayesian optimization process for target roughness: (**a**) convergence of prediction error during BO iterations; (**b**) distribution of sampled parameter combinations and the optimal point in the parameter space.

Based on the RF prediction result and the experimental parameter bounds, Bayesian optimization was used to search for process parameters corresponding to a target Ra of 5 μm. The decision variables were bounded at 200–500 W for laser power, 1–5 mm/s for scanning speed, 0–50% for spot overlap ratio, and 1–5 for scan number. The BO search used a Gaussian-kernel probabilistic surrogate and an expected-improvement acquisition function. As shown in Figure 11, the optimization process gradually concentrated the search near the low-error region. After 50 iterations, the relative error between the predicted roughness and the target roughness converged to 0.0231%.

The optimization trajectory can be divided into an initial exploration stage and a later refinement stage. During the early iterations, the current error fluctuated strongly because the acquisition function sampled uncertain regions to construct a global response surface. As more observations accumulated, the search gradually concentrated in a region characterized by high power, moderate-to-low scanning speed, high overlap, and a large scan number. This region is physically reasonable because it provides sufficient remelting energy, adequate spreading time, strong inter-track coupling, and repeated thermal leveling.

The optimized parameter combination was 500 W laser power, 2.57 mm/s scanning speed, 48.09% overlap, and five scans. The predicted roughness under this condition was 5.02 μm. Compared with the single-factor tests, this result indicates that the target roughness was not determined by one parameter alone. Instead, the optimized condition balanced sufficient power, moderate residence time, high track overlap, and repeated remelting. This is consistent with the experimental observation that power, scanning speed, overlap ratio, and scan number jointly determine whether surface defects are melted, redistributed, and finally solidified into a smoother sidewall.

### 3.6. Experimental Validation

**Figure 12:**
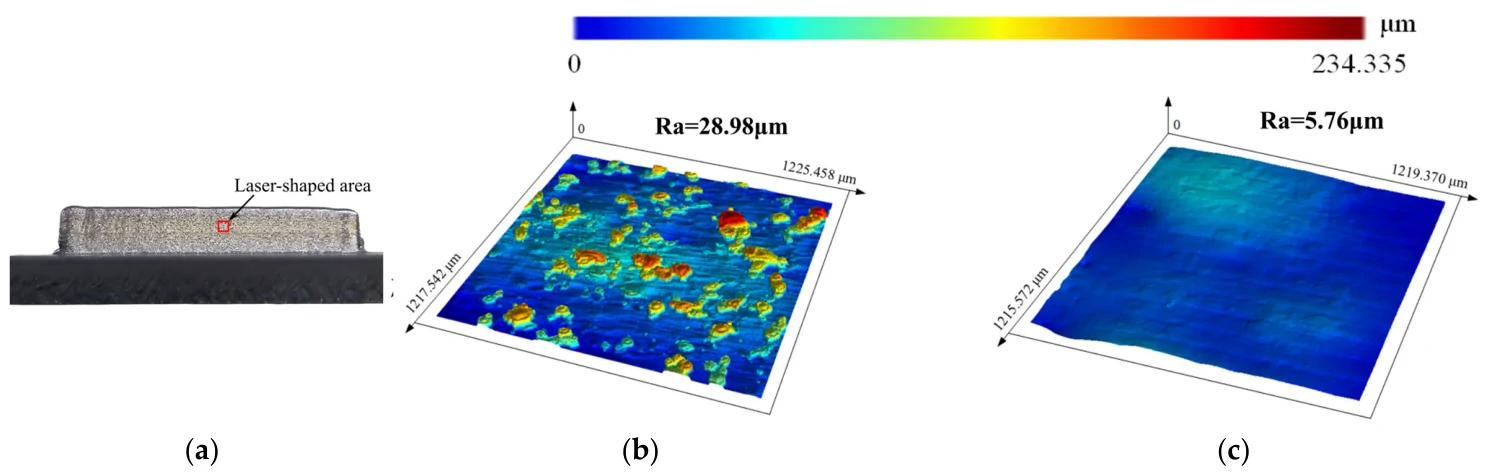
Experimental validation under optimized reshaping parameters: (**a**) laser-reshaped area on the validation specimen; (**b**) sidewall height map before reshaping; (**c**) sidewall height map after reshaping.

Figure 12 compares the sidewall morphology before and after reshaping under the optimized parameter combination. Before reshaping, the sidewall contained a large number of adhered particles and irregular protrusions, and the measured Ra was 28.98 μm. After square-spot laser reshaping, the surface became smoother and more uniform, and the measured Ra decreased to 5.76 μm. This corresponds to an 80.1% reduction in roughness.

The experimental value was close to the BO-predicted value of 5.02 μm. The absolute deviation was 0.76 μm, and the relative error was 14.7%. Considering the thermal fluctuations of laser additive manufacturing, the discrete scan number, and the sensitivity of surface remelting to local morphology, this deviation is acceptable for process-window design. The validation confirms that the proposed experimental-ML-BO framework can identify practical laser reshaping parameters for a prescribed sidewall roughness target.

The final surface morphology also supports the numerical result. Before reshaping, the sidewall height map contained many discrete high peaks associated with adhered particles and irregular protrusions. After reshaping under the optimized condition, these rough features largely disappeared and were replaced by a smoother and more uniform surface. The remaining error between prediction and experiment is mainly attributed to local variability of the initial sidewall morphology, experimental operation error, and rounding of the discrete scan number.

## 4. Discussion

The experimental results show that sidewall roughness control in LDED Ti-6Al-4V thin-walled structures is governed by the balance between sufficient remelting and excessive thermal accumulation. This agrees with previous laser polishing and laser remelting studies, which reported that surface peaks must be remelted and redistributed without producing excessive melt-pool instability or thermal damage [7,8,9,10,11,12,22,23,24,25]. Insufficient heat input leaves adhered particles and layer steps only partly melted, whereas excessive or poorly distributed heat input can generate remelted undulations, local surface damage, or dimensional degradation. The square-spot beam is advantageous because its flat-top energy distribution provides a more uniform heat input over the processed area than a beam with a strong central peak. This is especially important for multi-track sidewall reshaping, where track overlap determines whether adjacent paths merge smoothly or leave visible boundary grooves [13].

The role of each process variable should therefore be interpreted through molten-layer dynamics rather than through isolated single-parameter effects. Laser power determines whether the surface reaches a temperature high enough to remelt adhered particles and step edges. Scanning speed governs how long the molten layer remains fluid before rapid solidification. Overlap ratio determines whether neighboring tracks remain thermally independent or are mutually remelted, which is consistent with the reported influence of laser-track overlap on final surface quality [13]. Scan number controls how many times residual roughness features experience a remelting-leveling cycle. Accordingly, the minimum roughness cannot be obtained by maximizing any single variable; it requires a coupled processing window that provides sufficient energy and spreading time without causing overmelting.

Laser power was identified as the dominant factor in both the single-factor experiments and the SHAP interpretation. This dominance is physically reasonable because power directly controls the peak temperature, remelted-layer thickness, molten-pool width, and melt viscosity during surface reshaping. When power is too low, adhered particles and layer steps remain only partially melted; when power is sufficient, capillary-driven flow and surface-tension leveling can redistribute molten material into valleys before solidification. Scanning speed, overlap ratio, and scan number modify the residence time, inter-track thermal accumulation, and repeated-remelting history, but their effects depend on whether the power first establishes a stable molten layer. In this sense, SHAP did not replace physical interpretation; it provided a model-based check that the learned parameter effects were consistent with heat-transfer and melt-flow behavior [17,20].

The RF model performed best for the present small nonlinear dataset. Unlike SVR, which tends to produce a globally smooth response, RF can capture local changes caused by discrete process levels and parameter interactions [18]. Compared with XGBoost, which is powerful but can be sensitive to individual samples when the dataset is small, RF also showed more stable error behavior in this dataset [19]. The 80-sample dataset is therefore considered adequate for building a local predictive model within the tested process window, especially because the model ranking, SHAP trends, single-factor experiments, and validation experiment were mutually consistent. However, this sample size is not sufficient for claiming universal generalization across all LDED systems or laser-reshaping configurations.

This point is important for applying machine learning to manufacturing parameter design. Recent reviews and case studies have emphasized that machine-learning models in additive manufacturing should be evaluated not only by numerical accuracy, but also by whether their feature effects and prediction trends are consistent with process physics [14,15,16,17,26,27]. In this work, the RF model was preferred not only because it achieved the highest R2 and the lowest RMSE, but also because its SHAP interpretation agreed with the single-factor experiments and its optimized recommendation was experimentally validated. The agreement among experiment, prediction, interpretation, and validation reduces the likelihood that the BO search was driven only by overfitting to a small dataset.

The BO result further demonstrates the value of coupling a forward roughness predictor with inverse parameter design. Bayesian optimization is suitable for process-window search because it can use a surrogate model and acquisition function to balance exploitation of promising regions with exploration of uncertain regions [21,28]. In the present validation, the target roughness was set to 5 μm, the predicted result was 5.02 μm, and the validation experiment yielded 5.76 μm. The absolute deviation of 0.76 μm should be interpreted together with the RF prediction error (RMSE = 1.760 μm). This means that the predicted optimum is not a single error-free point, but a practical parameter recommendation with model uncertainty. If the RF prediction error changes locally, the BO optimum may shift, particularly because the selected power and scan number were near upper search bounds.

Compared with a conventional Gaussian beam, the square flat-top beam is expected to reduce center overheating and edge under-melting under comparable scanning conditions because the heat input is distributed more uniformly across the effective spot area. This explains why the square-spot strategy is suitable for sidewall reshaping where adjacent tracks must merge smoothly. Nevertheless, the present study did not include a fully equivalent Gaussian-beam polishing control under the same line energy and scanning geometry. Therefore, the practical advantage of the square spot is supported by energy-distribution analysis and reshaping results in this work, but a direct quantitative Gaussian-versus-square comparison should be included in future studies.

The current single-objective optimization focused on minimizing surface roughness. For industrial use, roughness should be optimized together with dimensional accuracy, wall-thickness retention, geometric distortion, oxidation control, heat-affected-zone size, and possibly productivity. Excessive remelting may improve Ra while degrading sidewall thickness or local dimensional integrity, especially in thin-walled structures with low stiffness. Therefore, future BO implementation should be extended to a multi-objective framework that constrains geometric deviation and thermal damage while reducing roughness.

Repeated laser scanning may also influence microstructure, residual stress, oxidation behavior, and mechanical properties of the reshaped Ti-6Al-4V surface. These effects were outside the scope of the present roughness-control study and were not quantified here. Because Ti-6Al-4V is sensitive to thermal cycling and oxygen pickup, future work should combine surface roughness measurement with microstructural characterization, oxygen-content analysis, residual-stress measurement, and mechanical testing before the process is transferred to load-bearing components.

The practical implementation of the proposed workflow can follow a staged route. First, a small calibration dataset should be established for a specific LDED system, wall geometry, powder batch, and square-spot reshaping setup. Second, an interpretable model should be trained and checked against physical trends using SHAP or similar tools. Third, BO can be used to recommend a small number of candidate parameter sets for confirmatory experiments. Finally, the process window should be locked only after roughness, dimensional accuracy, and metallurgical quality meet the relevant industrial tolerance requirements.

Overall, the optimized parameter set should be interpreted as a validated condition within the present material, wall geometry, equipment configuration, and parameter bounds. It should not be generalized directly to different LDED systems, laser powers, spot sizes, material batches, or wall dimensions without additional calibration. The transferable contribution is the experimental-ML-BO workflow rather than a universal numerical parameter set.

## 5. Conclusions

Square-spot laser surface reshaping combined with machine learning-assisted parameter design provides an effective route for controlling sidewall roughness in LDED Ti-6Al-4V thin-walled structures. In this study, a complete chain was established from experimental parameter screening to prediction, interpretation, inverse design, and experimental validation.

1. The single-factor experiments showed that sidewall roughness was governed by the coupled effects of laser power, scanning speed, spot overlap ratio, and scan number. Higher power, appropriate residence time, sufficient track overlap, and repeated remelting all promoted molten-metal leveling and roughness reduction.
2. Among the three prediction models, RF provided the best overall performance, with R^2^ = 0.940 and RMSE = 1.760 μm. SHAP interpretation further confirmed that laser power made the largest contribution to roughness prediction, followed by scanning speed and spot overlap ratio, consistent with the experimental trends.
3. BO successfully inversely determined a parameter set for a target roughness of 5 μm. The optimized condition of 500 W, 2.57 mm/s, 48.09% overlap, and five scans reduced Ra from 28.98 μm to 5.76 μm, corresponding to an 80.1% reduction in roughness, and the absolute deviation from the predicted value was 0.76 μm.

## Figures and Tables

**Table 1:** Single-factor square-spot laser surface reshaping parameters.

No.	Laser Power (W)	Scanning Speed (mm/s)	Spot Overlap Ratio (%)	Scan Number
1	200	2	0	1
2	300	2	0	1
3	400	2	0	1
4	500	2	0	1
5	500	1	0	1
6	500	2	0	1
7	500	3	0	1
8	500	4	0	1
9	500	2	0	1
10	500	2	10	1
11	500	2	20	1
12	500	2	30	1
13	500	2	0	1
14	500	2	0	2
15	500	2	0	3
16	500	2	0	4

**Table 2:** Dataset of process parameters and sidewall roughness.

No.	Laser Power (W)	Scanning Speed (mm/s)	Spot Overlap Ratio (%)	Scan Number	Ra (μm)
1	200	2	30	3	10.14
2	200	4	20	4	11.60
3	200	4	0	4	12.85
4	200	2	10	2	14.04
5	200	2	30	2	20.85
6	200	1	30	2	21.82
7	200	3	20	3	22.00
8	200	4	30	4	22.05
9	200	1	10	1	22.39
10	200	3	0	1	22.82
11	200	3	0	3	23.89
12	200	2	30	1	24.05
13	200	1	0	1	24.20
14	200	3	20	2	24.41
15	200	4	10	1	24.74
16	200	2	20	1	25.86
17	200	2	0	1	27.20
18	300	1	10	4	7.68
19	300	3	20	3	8.11
20	300	4	30	3	9.97
21	300	2	20	2	10.78
22	300	2	10	4	11.34
23	300	3	30	1	11.83
24	300	1	0	1	12.07
25	300	4	20	2	12.54
26	300	4	30	1	12.66
27	300	3	0	2	13.03
28	300	4	0	4	13.54
29	300	1	0	3	13.82
30	300	2	0	4	14.60
31	300	2	0	1	17.11
32	300	1	10	3	17.92
33	300	1	0	4	21.74
34	300	2	30	1	23.06
35	400	1	30	3	6.18
36	400	4	10	1	6.63
37	400	1	20	1	7.02
38	400	2	10	3	7.96
39	400	2	30	3	9.24
40	400	1	20	2	9.39
41	400	3	30	3	10.38
42	400	1	10	3	11.19
43	400	3	0	4	11.65
44	400	1	20	4	11.85
45	400	3	0	2	12.71
46	400	3	20	4	13.41
47	400	1	10	1	15.07
48	400	2	30	2	16.26
49	400	4	30	3	18.06
50	400	2	0	1	20.16
51	400	3	20	2	21.06
52	500	1	30	2	1.82
53	500	2	20	1	2.03
54	500	2	30	3	3.16
55	500	4	20	4	3.54
56	500	2	10	4	3.58
57	500	1	10	2	4.99
58	500	2	30	1	4.03
59	500	2	30	2	4.37
60	500	2	20	4	4.64
61	500	2	0	4	4.76
62	500	1	20	2	3.73
63	500	2	0	3	5.12
64	500	3	10	3	5.33
65	500	4	30	4	5.85
66	500	4	0	3	5.98
67	500	4	0	1	6.20
68	500	3	10	4	6.24
69	500	3	10	2	6.99
70	500	4	0	2	7.20
71	500	3	0	1	7.39
72	500	2	20	2	7.40
73	500	2	0	2	9.37
74	500	3	30	3	10.28
75	500	2	0	1	24.30
76	500	1	10	1	23.67
77	500	1	0	1	12.31
78	500	2	10	1	17.37
79	500	2	10	2	24.75
80	500	1	0	2	22.11

**Table 3:** Parameter bounds for Bayesian optimization.

Process Parameter	Lower Bound	Upper Bound
Laser power (W)	200	500
Scanning speed (mm/s)	1	5
Spot overlap ratio (%)	0	50
Scan number	1	5

## Data Availability

The original contributions presented in this study are included in the article. Further inquiries can be directed to the corresponding author.

## Abbreviations

The following abbreviations are used in this manuscript:

LDED	Laser Directed Energy Deposition
RF	Random Forest
SVR	Support Vector Regression
SHAP	SHapley Additive exPlanations
BO	Bayesian Optimization
CLSM	Confocal Laser Scanning Microscope
XGBoost	eXtreme Gradient Boosting
LHS	Latin Hypercube Sampling
RMSE	Root Mean Square Error
MAE	Mean Absolute Error
